# Efficacy and safety of dapagliflozin, a sodium glucose cotransporter 2 (SGLT2) inhibitor, in diabetes mellitus

**DOI:** 10.1186/s12933-015-0297-x

**Published:** 2015-10-17

**Authors:** Paola Fioretto, Andrea Giaccari, Giorgio Sesti

**Affiliations:** Department of Medicine, University of Padua, Via Giustiniani 2, 35128 Padua, Italy; EndoMetabolic Diseases Unit, Policlinico Gemelli, Università Cattolica del Sacro Cuore, Largo A. Gemelli 8, 00168 Rome, Italy; Department of Medical and Surgical Sciences, University Magna-Græcia of Catanzaro, Viale Europa, 88100 Catanzaro, Italy

**Keywords:** Antidiabetic drugs, Dapagliflozin, Glycosylated hemoglobin, Glycemic control, Sodium-glucose cotransporter 2 inhibitors, Type 1 diabetes mellitus, Type 2 diabetes mellitus, Weight reduction

## Abstract

Although antidiabetic agents have been developed to target one or more of the core defects of type 2 diabetes mellitus (T2DM), many patients do not achieve glycemic goals. Inhibition of the sodium-glucose cotransporter 2 (SGLT2) induces glycosuria, reduces glucose toxicity and improves insulin sensitivity and β-cell function. As the mechanism of action of SGLT2 inhibitors is different from other agents and completely insulin-independent, the use of these drugs might potentially be efficacious alone or in combination with any other antidiabetic drug, including insulin. Dapagliflozin is a highly selective and reversible SGLT2 inhibitor approved for use in adult patients with T2DM as monotherapy in patients intolerant of metformin or as adjunctive therapy in patients inadequately controlled on existing antidiabetic medications, including insulin. A literature search conducted using PubMed identified key publications related to the use of dapagliflozin in the treatment of patients with diabetes mellitus. No date limits were applied. This review focuses on the safety and efficacy of this SGLT2 inhibitor. Dapagliflozin produces dose-related reductions in glycosylated hemoglobin (HbA_1c_) as monotherapy and as add-on to other antidiabetic agents, with significant reductions in body weight. Hypoglycemia is uncommon. Preliminary data from a phase 2 pharmacokinetic/pharmacodynamic study suggest that dapagliflozin may also improve glycemic control in patients with type 1 diabetes mellitus. Clinical trials published to date show that dapagliflozin is safe and effective as monotherapy or as an add-on to insulin or oral antidiabetic agents in patients with T2DM.

## Background

The pathophysiology of type 2 diabetes mellitus (T2DM) is complex and multifaceted. The core defects of T2DM include quantitative and qualitative β-cell dysfunction, peripheral (skeletal muscle) insulin resistance, and elevated glucose production in the liver, as well as increased lipolysis when obesity is present. However, it is becoming accepted that other known mechanisms, including increased glucagon, decreased incretin effect, increased glucose reabsorption in the kidneys and some neurotransmitter dysfunction, are also involved in the pathophysiology of T2DM [1].

The currently available antidiabetic agents have been developed to target one or more of the underlying defects or processes involved in T2DM [2]. Generally, glycemic control in patients with T2DM is poor, with only approximately 53 % of patients achieving glycemic goals with their current treatment regimen [3].

However, even in patients with good glycemic control, the progressive nature of T2DM means that most patients will eventually require multiple antidiabetic medications to manage their disease [4]. As a result, there remains a need for new drug development in the field.

This review discusses one of the most recently discovered classes of antidiabetic agents, the inhibitors of the sodium-glucose cotransporter 2 (SGLT2). SGLT2 inhibitors currently approved or under investigation include dapagliflozin, canagliflozin, empagliflozin and ipragliflozin; this manuscript will focus on the efficacy and safety of the highly selective and reversible SGLT2 inhibitor dapagliflozin, primarily in patients with T2DM.

## Search strategy

A literature search was conducted using the PubMed database to identify key papers related to human studies of dapagliflozin in the treatment of patients with diabetes mellitus. Various combinations of key terms, including “type 2 diabetes”; “type 1 diabetes”; “SGLT2” or “sodium-coupled glucose cotransporter 2” “monotherapy”; “add-on or combination”; and “dapagliflozin”, were used, with no date limits applied. The search focused on clinical trials, relevant sub-studies of clinical trials identified, meta-analyses and systematic reviews. Additional papers were included from the reference lists of relevant articles sourced from the search. Recent guidelines and consensus documents were also considered for inclusion. The initial search was updated to include articles published up to March 27, 2015.

## The role of the kidney in glucose reabsorption

The kidneys have a major role in glucose regulation in humans; under normal circumstances, over 99 % of the glucose filtered by the glomeruli is reabsorbed in the proximal tubules [5, 6], such that virtually no glucose is excreted in the urine. Renal glucose reabsorption is achieved through the action of the SGLT family of protein transporters, primarily SGLT1 and SGLT2 [6]. These proteins transport glucose across the membranes of the proximal tubule epithelial cell in an active process that involves sodium transport, facilitated by the sodium gradient between the tubule and the cell, which supports secondary active co-transport of glucose. Glucose then passively diffuses into the intercellular space mainly via the GLUT2 (glucose transporter 2), a member of the GLUT family of proteins [6, 7].

In individuals without T2DM, SGLT2 is responsible for the majority (80–90 %) of renal glucose reabsorption [6, 8]. When the concentration of glucose in plasma exceeds that which can be reabsorbed via the SGLT proteins, the excessive amount of glucose is excreted in the urine [7, 9]. Furthermore, data from animal models of diabetes and from preclinical human models suggest that the hyperglycemic state in T2DM is associated with significantly increased expression of SGLT2 and GLUT2 proteins, together with an increased level of renal glucose reabsorption [7, 10]. Therefore, SGLT2 is a novel therapeutic target for antidiabetic therapy.

## The role of glycosuria in glucotoxicity

The harmful metabolic effects of chronic hyperglycemia associated with the two core defects of T2DM, insulin resistance and partial β-cell failure, can be described as “glucose toxicity”, which contributes in turn to further progression of β-cell dysfunction in a vicious cycle of glucose toxicity-induced pathogenesis [11, 12]. Thus, tight glycemic glucose control is, in part, considered essential to reverse or slow the glucotoxic effects of chronic hyperglycemia on the β-cells [4].

Glycosuria occurs when the maximal reabsorptive capacity of the glucose transport system in the kidney becomes saturated, above or just below the theoretical plasma glucose threshold of approx 11 mmol/L (198 mg/dL); both the threshold and the transport maximum of glucose are higher in patients with diabetes mellitus [8]. Conversely, induction of glycosuria without affecting other metabolic parameters has been shown to reduce elevated plasma glucose levels, supporting the hypothesis that glucose toxicity contributes to β-cell dysfunction in patients with T2DM and that reversing glucose toxicity via “therapeutic glycosuria” may improve insulin sensitivity and β-cell function [11, 13, 14].

## Sodium-glucose cotransporter 2 inhibitors

**Fig. 1:**
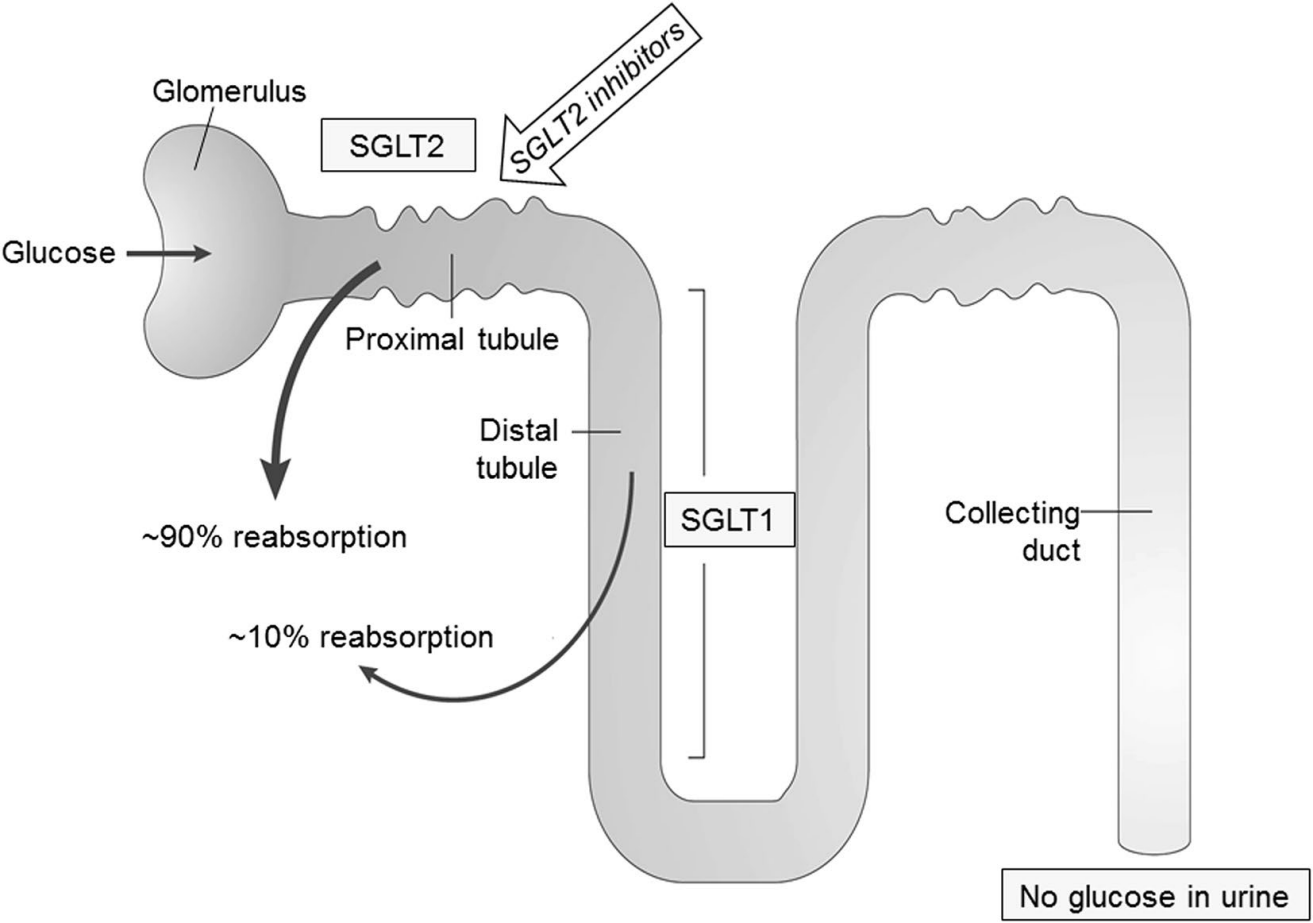
Glucose reabsorption by the normal kidney, showing the site of action of sodium-glucose cotransporter (SGLT) 2 inhibitors. Modified with permission from Chao and Henry [7]

Renal tubule regulation of glucose reabsorption by the kidney in the non-diabetic individual is shown in Fig. 1. Inhibition of SGLT2 results in a lowering of the threshold for renal glucose excretion and an increase in urinary glucose excretion, with an associated reduction in plasma glucose levels and the potential to decrease glucose toxicity with chronic administration [7, 15–17]. The action of SGLT2 inhibitors is glucose-dependent, becoming negligible when plasma glucose concentration drops below 90/mg/dL, so the risk of hypoglycemia is lower than with insulin-dependent antidiabetic drugs [18].

Evidence suggests that glycosuria induced by SGLT2 inhibitors can also significantly improve β-cell insulin secretion and insulin sensitivity in peripheral tissues, associated with a reduction in plasma glucose concentration [13, 14, 16, 17, 19]. In addition, SGLT2 inhibitors as a class offer the clinical benefits of promoting body weight loss and produce a modest reduction in both systolic and diastolic blood pressure [16–18, 20–26].

The mechanisms of action of the blood pressure lowering effects of SGLT2 inhibitors are not fully understood. However, they are likely due to a mild osmotic diuretic effect in association with reductions in body weight and increased hematocrit, although it is possible that there may be a contribution from local inhibition of the renin-angiotensin system secondary to enhancement of sodium delivery to the juxtaglomerular apparatus [20, 23, 27]. Orthostatic hypotension is not increased with SGLT2 inhibitors.

Weight loss associated with SGLT2 inhibitors is biphasic, with an initial reduction in total body weight which can be attributed to fluid loss and a subsequent gradual continuous reduction resulting from increased urinary glucose excretion with associated loss of calories [14, 17, 18, 22]. Body composition assessment using dual-energy X-ray absorptiometry and magnetic resonance imaging analysis in patients with T2DM treated with dapagliflozin showed that the reduction in total body weight was predominantly via reductions in total body fat mass, visceral adipose tissue and subcutaneous adipose tissue volume [28].

The effects of SGLT2 inhibitors on hyperglycemia, body weight, and blood pressure suggest a favorable effect on cardiovascular risk factors. However, data on long-term effects on major cardiovascular outcomes, including myocardial infarction, stroke, and other vascular endpoints, are for the most part still under investigation, with results expected over the next few years [16, 22, 26]. Results of studies available to date show that empagliflozin ameliorated cardiovascular injury, coronary artery remodeling, and vascular dysfunction in a T2DM model in obese mice, suggesting a possible role in preventing diabetic macrovascular and microvascular complications [29], and did not prolong the QT interval in healthy volunteers [30]. Empagliflozin was also associated with a decline in arterial stiffness, a marker for renal and cardiovascular clinical outcomes, in young patients with type 1 diabetes mellitus (T1DM) [21]. While this review was under revision, Zinman et al. published in the *New England Journal of Medicine* the results of the EMPA-REG OUTCOME Study [31], demonstrating the achievement of the pre-defined primary endpoint (3-point reduction of MACE). Among the different effects of empagliflozin (and of all SGLT2 inhibitors), none appeared to be specifically responsible for the significant reduction of cardiovascular events. This suggests that such CV protection might be assumed as a class effect, but firm evidence for this assumption will not be available before 2017–2019 (https://clinicaltrials.gov/ct2/show/record/NCT01032629, https://clinicaltrials.gov/ct2/show/NCT01730534).

SGLT2 inhibitors as a class have generally been shown to be as effective as other antidiabetic agents, with a good safety profile [18, 32]. Data from phase II/III clinical trials, pooled analyses of randomized controlled trials, and systematic reviews show that SGLT2 inhibitors are as effective as other antidiabetic agents such as metformin, sulfonylureas, or dipeptidyl peptidase-4 (DPP-4) inhibitors in head-to-head trials, and may offer better long-term glucose-lowering efficacy [16–18, 24, 32, 33].

The risk of hypoglycemia is lower with SGLT2 inhibitors than with conventional antidiabetic agents, but there is a higher risk of genital infections (mostly mycoses on the external genitals) [16–18, 22, 24, 26, 32]. The effects of SGLT2 inhibitors on macrovascular and microvascular outcomes are yet to be determined in human studies; however, adding SGLT2 inhibitors, and specifically dapagliflozin, to the standard of care was recently projected to reduce cardiovascular and microvascular complications associated with T2DM, in a human model study using simulation methodology [34].

The efficacy of SGLT2 inhibitors is influenced by the level of hyperglycemia and renal function [18, 22, 27]. Patients with substantial levels of hyperglycemia have a greater level of urinary glucose excretion and plasma glucose reduction. Conversely, patients with a lower glomerular filtration rate have a lower level of urinary glucose excretion, which is likely to lead to a lesser glucose-lowering effect [35]. Long-term study of patients with T2DM and moderate renal impairment showed that, although dapagliflozin reduced weight and blood pressure, glycemic control was not improved.

## Dapagliflozin in type 2 diabetes mellitus

Dapagliflozin is indicated for patients aged 18 years and over with T2DM for use as monotherapy to improve glycemic control in patients with inadequate glycemic control who are intolerant to metformin, and as an adjunct to diet and exercise in combination with other glucose-lowering medicinal agents in patients inadequately controlled on existing antidiabetic medications, including insulin [36].

In patients with moderate renal impairment [creatinine clearance <60 mL/min or estimated glomerular filtration rate (eGFR) <60 mL/min/1.73 m^2^] dapagliflozin has been shown to be less effective. Therefore, dapagliflozin is not recommended for use in patients with moderate to severe renal impairment. Such limitations have not been introduced for the presence of side effects, but in recognition of reduced efficacy in this patient population.

The efficacy and safety of dapagliflozin as monotherapy and as add-on/combination therapy with existing antidiabetic treatment in patients with diabetes mellitus has been established in a series of phase II and III trials in the clinical development program and is supported by five recently published comprehensive reviews or meta-analyses [37–41]. It should be noted that, while different dosages of dapagliflozin were evaluated in the above-mentioned trials, the only dosage currently approved in Europe is 10 mg in oral administration once daily.

### Efficacy as monotherapy

Dapagliflozin has been shown to be effective in several large (*n* *>* 261), randomized, double-blind, placebo-controlled studies of 12 or 24 weeks’ duration in treatment-naïve patients with T2DM [25, 42–46]. Dapagliflozin was evaluated at doses ranging from 1 to 50 mg once daily when added to background diet and exercise [42–44]; compared with metformin monotherapy [45, 47]; or administered as monotherapy in both treatment-experienced and treatment-naïve patients [25].

Dapagliflozin monotherapy demonstrated dose-related reductions from baseline in glycosylated hemoglobin (HbA_1c_) in all dapagliflozin groups, ranging from mean reductions of −0.55 to −1.45 % overall [25, 42–46] (Table 1). Statistically significant reductions in fasting plasma glucose (FPG) and body weight for dapagliflozin versus placebo or metformin were observed. Hypoglycemia was uncommon in dapagliflozin-treated patients; genital and urinary tract infections were more common in dapagliflozin groups than in controls.

**Table 1:** Glycemic efficacy of dapagliflozin when used as monotherapy in patients with type 2 diabetes mellitus

Study	Intervention	Mean change from baseline HbA_1c_, %
List et al. [45]	Placebo (*n* = 54)	−0.18
	Dapagliflozin 2.5 mg (*n* = 59)	−0.71**
	Dapagliflozin 5 mg (*n* = 58)	−0.72**
	Dapagliflozin 10 mg (*n* = 47)	−0.85**
	Dapagliflozin 20 mg (*n* = 59)	−0.55*
	Dapagliflozin 50 mg (*n* = 56)	−0.90**
	Metformin XR 750/1500 mg (*n* = 56)	−0.73
Ferrannini et al. [43]	Placebo (*n* = 75)	−0.23
	Dapagliflozin 2.5 mg a.m. (*n* = 65)	−0.58
	Dapagliflozin 5 mg a.m. (*n* = 64)	−0.77**
	Dapagliflozin 10 mg a.m. (*n* = 70)	−0.89^†^
	Dapagliflozin 2.5 mg evening (*n* = 67)	−0.83
	Dapagliflozin 5 mg evening (*n* = 68)	−0.79
	Dapagliflozin 10 mg evening (*n* = 76)	−0.79
Bailey et al. [42]	Placebo (*n* = 68)	0.02
	Dapagliflozin 1 mg (*n* = 72)	−0.68^†^
	Dapagliflozin 2.5 mg (*n* = 74)	−0.72^†^
	Dapagliflozin 5 mg (*n* = 68)	−0.82^†^
Henry et al. [47]	Dapagliflozin 5 mg (*n* = 203)	−1.19
	Metformin XR 2000 mg (*n* = 201) (study 1)	−1.35
	Dapagliflozin 10 mg (*n* = 219)	−1.45
	Metformin XR 2000 mg (*n* = 208) (study 2)	−1.44
Kaku et al. [46]	Placebo (*n* = 54)	0.37
	Dapagliflozin 1 mg (*n* = 59)	−0.12**
	Dapagliflozin 2.5 mg (*n* = 56)	−0.11**
	Dapagliflozin 5 mg (*n* = 58)	−0.37**
	Dapagliflozin 10 mg (*n* = 52)	−0.44**
Ji et al. [44]	Placebo (*n* = 132)	−0.29
	Dapagliflozin 5 mg (*n* = 128)	−1.04
	Dapagliflozin 10 mg (*n* = 133)	−1.11
Kaku et al. [25]	Placebo (*n* = 87)	−0.06
	Dapagliflozin 5 mg (*n* = 86)	−0.41^†^
	Dapagliflozin 10 mg (*n* = 88)	−0.45^†^

*a.m.* morning, *HbA*_*1c*_, glycosylated hemoglobin

* *P* < 0.01 vs. placebo, ** *P* < 0.001 vs. placebo, ^†^ *P* < 0.0001 vs. placebo

Dapagliflozin monotherapy was generally as effective as metformin monotherapy [47, 48], and the glycemic efficacy and reduction in weight of dapagliflozin monotherapy seen in short-term studies is maintained in the long term (52–102 weeks) [33, 48].

In addition, the data from the studies in Asian patients with T2DM suggest that dapagliflozin is effective in that population [25, 33, 48]. More recent data support similar efficacy of dapagliflozin in white, black and Hispanic patients, suggesting its use may be beneficial irrespective of race [49].

### Efficacy as add-on or combination therapy

A number of randomized, double-blind, placebo-controlled studies in patients with T2DM have evaluated the efficacy of dapagliflozin as adjunctive therapy with metformin [28, 47, 50–57] and insulin [58–61].

Glycemic efficacy and body weight data from key studies of dapagliflozin add-on/combination therapy with metformin or insulin are presented in Table 2. Dapagliflozin is associated with clinically and statistically significant improvements in glycemic control and reductions in body weight, compared with placebo, when added to concurrent metformin or insulin therapy. Mean adjusted changes from baseline HbA_1c_ ranged from −0.39 to −0.96 % in the primary studies, compared with +0.02 to −0.39 for placebo (Table 2). Mean adjusted changes from baseline body weight with dapagliflozin ranged from −0.90 to −5.05 kg in the primary studies, compared with +0.4 to −1.55 kg for placebo (Table 2). Changes in body weight associated with dapagliflozin primarily result from changes in fat mass (mediated by glycosuria), rather than fluid: according to Bolinder et al., after 102 weeks reductions in total body fat mass were −2.80 kg (95 % CI −3.67, −1.93) in the dapagliflozin group, compared with −1.46 kg (95 % CI −2.25, −0.68) in the placebo group [52].

**Table 2:** Glycemic efficacy of dapagliflozin when used in combination therapy in patients with type 2 diabetes mellitus

Study	Intervention	Mean change from baseline HbA_1c_, %		Mean change from baseline bodyweight (kg)	
		To end of main study	To end of extension	To end of main study	To end of extension
Metformin studies^a^					
Bailey et al. [50]	Placebo (*n* = 137; 73)	−0.30	0.02	−0.9	1.4
Bailey et al. [51] (24 weeks; 102 weeks)					
	Dapagliflozin 2.5 mg (*n* = 137; 82)	−0.67^†^	−0.48^†^	−2.2^‡^	−1.1^‡^
	Dapagliflozin 5 mg (*n* = 137; 89)	−0.70^‡^	−0.58^‡^	−3.0^‡^	−1.7^‡^
	Dapagliflozin 10 mg (*n* = 135; 95)	−0.84^‡^	−0.78^‡^	−2.9^‡^	−1.7^‡^
Nauck et al. [53]	Dapagliflozin ≤10 mg (*n* = 400; 161)	−0.52	−0.10	−3.2^‡^	−4.0
Del Prato et al. [56] (52 weeks; 4 years)					
	Glipizide ≤20 mg (*n* = 401; 141)	−0.52	0.2	1.4	1.1
Bolinder et al. [28]	Placebo (*n* = 91; 49)	−0.10	−0.12	−0.88^‡^	−2.12
Bolinder et al. [52] (24 weeks; 2 years)					
	Dapagliflozin 10 mg (*n* = 89; 60)	−0.39^‡^	−0.30	−2.96	−4.54
Matthaei et al. [57]	Placebo (*n* = 108)	−0.17	–	−0.6	–
	Dapagliflozin 10 mg (*n* = 108)	−0.86^‡^	–	−2.7^‡^	–
Schumm-Draeger et al. [55]	Placebo (*n* = 101)	−0.30	–	−1.04	–
	Dapagliflozin 2.5 mg bd (*n* = 100)	−0.52*	–	−2.84^‡^	–
	Dapagliflozin 10 mg bd (*n* = 99)	−0.65^‡^	–	−3.2^‡^	–
	Dapagliflozin 10 mg od (*n* = 99)	−0.59^†^	–	−2.76^‡^	–
Insulin studies^b^					
Zhang et al. [60]	Placebo (*n* = 49)	−0.20	–	−0.95	–
Intensive insulin therapy ± insulin sensitizers at baseline	(patients with early-stage disease)				
	Dapagliflozin 10 or 20 mg (*n* = 102)	−0.70^†^ (10 mg)	–	−2.00 (10 mg)	–
	(patients with early-stage disease)	−0.50^†^ (20 mg)		−2.50 (20 mg)	
	Placebo (*n* = 14)	0	–	−1.55	–
	(patients with late-stage disease)				
	Dapagliflozin 10 or 20 mg (*n* = 44)	−0.60^†^ (10 mg)	–	−4.30 (10 mg)	–
	(patients with late stage disease)	−0.80^†^ (20 mg)		−5.05 (20 mg)	
Wilding et al. [58, 61]	Placebo (*n* = 193; 108)	−0.39	−0.43	0.4	1.8
Insulin therapy ± oral antihyperglycemic drugs at baseline (24 weeks; 2 years)					
	Dapagliflozin 2.5 mg (*n* = 202; 134)	−0.79^†^	−0.64*	−0.9^†^	−1.0^‡^
	Dapagliflozin 5 or 10 mg (*n* = 211; 129)	−0.89^†^	−0.82^†^	−1.0^†^	−1.0^‡^
	Dapagliflozin 10 mg (*n* = 194; 142)	−0.96^†^	−0.78^†^	−1.6^†^	−1.5^‡^

Key studies and their extensions

*bd* twice daily, *HbA*_*1c*_ glycosylated hemoglobin, *od* once daily

* *P* < 0.05 vs. placebo, ** *P* < 0.01 vs. placebo, ^†^ *P* < 0.001 vs. placebo, ^‡^ *P* < 0.0001 vs. placebo. Not all studies reported *P* values

^a^All patients were receiving metformin therapy

^b^All patients were receiving insulin therapy

In addition to placebo-controlled trials, dapagliflozin was compared with glipizide as add-on therapy to concurrent metformin [53, 54, 56]. At 52 weeks, glycemic efficacy was similar with dapagliflozin (change from baseline HbA_1c_ −0.52 %) and glipizide (−0.52 %), but dapagliflozin produced significant benefits in body weight reduction (−3.2 kg versus +1.2 kg, respectively; P < 0.0001), proportion of patients achieving ≥5 % body weight reduction (33.3 versus 2.5 %, respectively; P < 0.0001) and proportion of patients experiencing hypoglycemia (3.5 versus 40.8 %, respectively; P < 0.0001) [53]. Long-term data from the extension of this study to 104 weeks [54] and 208 weeks [56] showed that, compared with glipizide, dapagliflozin is associated with sustained glycemic efficacy, greater reductions in body weight and systolic blood pressure, and lower frequency of hypoglycemia (Table 2).

Dapagliflozin as an add-on or in combination with other antidiabetic agents also consistently produced reductions in blood pressure [37, 39, 40].

The results of longer-term extension studies demonstrate that the glycemic and body weight improvements with dapagliflozin are maintained after up to 4 years of follow-up. Mean adjusted changes from baseline HbA_1c_ at the end of the extension studies ranged from −0.30 to −0.82 % with dapagliflozin, compared with −0.10 to −0.43 for placebo (Table 2). Meanwhile, mean adjusted changes from baseline body weight ranged from −1.0 to −4.54 kg with dapagliflozin, compared with +1.8 to −2.12 kg for placebo (Table 2).

The effects of dapagliflozin have also been evaluated in add-on or combination with the dipeptidyl peptidase-4 inhibitor sitagliptin, the sulfonylurea glimepiride, and the thiazolidinedione pioglitazone. In patients inadequately controlled on sitagliptin with or without metformin, add-on therapy with dapagliflozin 10 mg provided additional clinical benefit without increasing hypoglycemia events [62].

In patients with inadequate glycemic control with glimepiride, dapagliflozin (at the 5 or 10 mg dose) significantly improved HbA_1c_ and significantly reduced body weight, compared with glimepiride alone [63]. At 24 weeks, the mean adjusted changes from baseline HbA_1c_ were −0.13 % for placebo versus −0.63 % with dapagliflozin 5 mg and −0.82 % with dapagliflozin 10 mg, respectively (both P < 0.0001 vs. placebo). Corresponding mean adjusted changes from baseline body weight were −0.72, −1.56 and −2.26 kg, respectively, for placebo, dapagliflozin 5 mg (P < 0.001 vs. placebo), and 10 mg (P < 0.0001 vs. placebo), respectively. There was a higher incidence of hypoglycemia in the dapagliflozin group (7.1–7.9 % vs. 4.8 %, respectively), as has been observed when dapagliflozin is added to sulfonylureas in other studies, and no patient discontinued treatment because of hypoglycemia.

Finally, in patients not adequately controlled on pioglitazone, dapagliflozin further lowered HbA_1c_ and lessened pioglitazone-associated weight gain [64]. At 24 weeks, the mean adjusted changes from baseline HbA_1c_ were −0.42 % for placebo versus −0.82 % with dapagliflozin 5 mg (P < 0.001 vs. placebo) and −0.97 % with dapagliflozin 10 mg (P < 0.0001 vs. placebo) respectively. Patients in the pioglitazone alone group gained significantly more body weight than those in the pioglitazone plus dapagliflozin groups. At 24 weeks, the mean adjusted changes from baseline body weight were +1.64, +0.09 and −0.14 kg, respectively, for placebo, dapagliflozin 5 mg, and dapagliflozin 10 mg (both P < 0.001 vs. placebo). By 48 weeks, patients in the pioglitazone alone group had gained a mean of 2.99 kg from baseline, compared with 1.35 kg for dapagliflozin 5 mg, and 0.69 kg for dapagliflozin 10 mg, respectively. However, dapagliflozin is not recommended for use in patients concomitantly treated with pioglitazone [65].

Longer-term extension phases of these trials demonstrate that reductions in HbA_1c_, FPG, and bodyweight are maintained during follow-up periods of up to 4 years [50, 52, 53, 56, 58, 61–64, 66].

A recent meta-analysis designed to evaluate whether dapagliflozin is synergistic with other antidiabetic agents without affecting body weight concluded that dapagliflozin in combination with conventional antidiabetic drugs (metformin, glimepiride, pioglitazone, and metformin/sitagliptin) improved glycemic control (the overall effect size was −0.52 %) and reduced weight gain in patients with T2DM (the effect size was −2.10 kg) [39]. Twelve randomized controlled trials with a total of 3986 participants were included in the glycemic control analysis (1996 dapagliflozin; 1990 controls), and 4008 in the body weight analysis (2005 dapagliflozin; 2003 controls). Follow-up durations ranged from 12 to 208 weeks [56].

Of interest, dapagliflozin 10 mg treatment significantly improved glycemic control and reduced body weight both in 151 early-stage and in 58 late-stage patients with T2DM, reflecting the usefulness of dapagliflozin as monotherapy in patients in the early stage of T2DM, and as add-on or combination therapy in late-stage patients on high doses of insulin plus oral insulin sensitizers [60].

To date, dapagliflozin in combination with glucagon-like peptide 1 (GLP-1) analogs is still being studied (https://clinicaltrials.gov/ct2/show/NCT02229396).

### Dapagliflozin and β-cell function

Improvements in β-cell function have been demonstrated for dapagliflozin using the homeostasis model assessment of β-cell function (HOMA β-cell) [40, 65], possibly resulting from a reduction in glucose toxicity. This hypothesis has been examined in a small study that demonstrated that lowering of plasma glucose concentration by dapagliflozin-induced glycosuria improved β-cell function and insulin resistance in patients with T2DM [13]. Importantly, these results demonstrate that the glucotoxic effect of chronic hyperglycemia on β-cell function in T2DM is, at least in part, reversible [13]. The authors theorized that, as dapagliflozin has not been shown to act directly on β-cell function, improvement in β-cell function was related to amelioration of hyperglycemia, that is, by reversing glucose toxicity [13]. Further investigation is warranted.

### Nephroprotection

In addition to improved glycemic control, reductions in serum uric acid levels and tubular glucose toxicity and attenuation of diabetes-related hyperfiltration suggest that SGLT2 inhibitors may be able to influence renal hemodynamics independently of glucose reduction [27, 40, 67, 68]. However, the effect of dapagliflozin and other SGLT2 inhibitors in slowing the development and progression of diabetic nephropathy is currently speculative, although preliminary data on the effect of dapagliflozin in patients already treated with renin-angiotensin system blockers have recently been presented, showing that dapagliflozin reduced albuminuria without increasing renal adverse events [69, 70].

### Safety and tolerability

The safety of dapagliflozin as monotherapy and as add-on therapy to existing antidiabetic treatment in patients with T2DM has been evaluated in multiple randomized controlled trials and has been assessed in five comprehensive reviews or meta-analyses [37–40, 71].

A pooled safety analysis of 12 placebo-controlled phase II/III clinical trials of up to 102 weeks duration that assessed dapagliflozin at doses of 2.5, 5, and 10 mg once daily in a total of more than 4000 patients has recently been published [71]. The analysis was supplemented by data from an active comparator trial, from a trial in patients with moderate renal impairment, and from five additional studies that were ongoing at the time of analysis, giving a total of 19 studies. In general, the analyses were performed for patients who received at least one dose of study medication during the double blind phase of the study. The patient populations in the pooled analyses were representative of the general population of patients with T2DM.

The majority (>90 %) of treatment-emergent adverse events (AEs) were mild-to-moderate in intensity, without a discernible dose relationship. One or more AEs were reported by 61.7 % of dapagliflozin recipients and 56.9 % of placebo recipients, and AEs were considered treatment-related in 17.3 % of dapagliflozin recipients and 13.3 % of the placebo group.

Hypoglycemia, urinary tract infections, vulvovaginitis/balanitis and related genital infections, back pain, polyuria, dysuria, and dyslipidemia were the most commonly reported AEs in the pooled dapagliflozin group (Table 3) [71]. In none of the studies did hypoglycemia lead to withdrawal, and it predominantly occurred when dapagliflozin was used with a sulfonylurea or insulin (Table 4). Similar proportions of dapagliflozin and placebo recipients reported serious AEs (3.7 vs. 3.3 %, respectively) and AEs resulting in study discontinuation (2.8 vs. 2.5 %, respectively). The tolerability profile demonstrated in the short-term studies was maintained consistently in patients receiving long-term dapagliflozin treatment.

**Table 3:** Incidence of adverse events (percent patients) reported in patients treated with dapagliflozin 5 mg, dapagliflozin 10 mg, or placebo

	Placebo	Dapagliflozin	
	(*N* = 1393)	5 mg (*N* = 1145)	10 mg (*N* = 1193)
Hypoglycemia^a^	7.0	10.9	10.2
Genital infection^b^	0.9	5.7	4.8
Urinary tract infection	3.7	5.7	4.3
Back pain	3.2	3.1	4.2
Polyuria	1.7	2.9	3.8
Dysuria	0.7	1.6	2.1
Dyslipidemia	1.5	2.1	2.5

Pooled data from 12 placebo-controlled phase II/III clinical studies. Modified with permission from Ptaszynska et al. [71]

^a^Major hypoglycemia requiring assistance or treatment and which, if left untreated may be life-threatening, occurred in 0.1 % of each of the dapagliflozin groups, and in 0.1 % of placebo recipients. Most hypoglycemic events were from add-on to insulin and add-on to sulfonylureas

^b^Vulvovaginitis/balanitis and related genital infections

**Table 4:** Incidence of hypoglycemia (percent patients) stratified by monotherapy and add-on therapies

	Placebo	Dapagliflozin	
		5 mg	10 mg
Placebo-controlled studies	7.0	10.9	10.2
Monotherapy studies	2.0	2.2	2.9
Add-on combination plus metformin	3.1	–	3.1
Plus pioglitazone	0.7	2.1	0
Plus a sulfonylurea	4.8	6.9	7.3
Plus insulin	35.0	45.3	42.3

Pooled data from 12 placebo-controlled phase II/III clinical studies. Data from Ptaszynska et al. [71]

No substantial adverse effects on serum electrolytes, liver function, or renal function were reported [71]. In general, there was a transient decrease in eGFR during the first weeks of dapagliflozin treatment, followed by a return to baseline levels or higher. Overall, there was no evidence of new or worsening renal impairment, acute nephrotoxicity, or progression of diabetic nephropathy in dapagliflozin-treated patients at up to 2 years [71]. Recent findings from routine clinical practice confirm that dapagliflozin therapy is not associated with nephrotoxicity [72].

Volume-related events, i.e. hypotension, dehydration and hypovolemia, were infrequent and none were serious, but occurred more often in the dapagliflozin group (0.8 %) compared with placebo (0.4 %) [71]. There was a higher risk of volume depletion events for dapagliflozin compared with placebo in patients also receiving loop diuretics (6.1 vs. 1.8 %, respectively). Numerically higher rates of dyslipidemia with dapagliflozin versus placebo have been reported, with small elevations in total cholesterol, low-density lipoprotein cholesterol and high-density lipoprotein cholesterol, and reductions in triglycerides [71]. There are some reports about patients on SGLT2 inhibitor treatment developing diabetic ketoacidosis [73]. Although there are no published reports of ketoacidosis specifically with dapagliflozin, it can be assumed that this is a class effect due to inappropriate prescriptions in patients with insufficient insulin (either endogenous or exogenous) and in which ketoacidosis has been masked by concomitant euglycaemia [74]. The US Food and Drug Administration and the European Agency of Medicine are currently investigating this issue through an extensive review of all available data and will consider whether any changes are needed in the way these medicines are used in the US and EU [75].

Although investigation into the cardiovascular effects of dapagliflozin and other SGLT2 inhibitors is ongoing, the cardiovascular safety of dapagliflozin appears to be favorable. An independently-adjudicated meta-analysis of data from >9000 patients with T2DM suggests that dapagliflozin does not increase cardiovascular risk in terms of MACE (major adverse cardiac events; a composite of cardiovascular death, non-fatal stroke, and non-fatal myocardial infarction) versus placebo or active control [76]. No adverse impact on cardiovascular safety of dapagliflozin treatment, compared with placebo, was recently reported even in high-risk patients with pre-existing cardiovascular disease and a history of hypertension [77]. Of interest, a multicenter, double blind, randomized, parallel group trial is underway (DECLARE-TIMI58 study; https://clinicaltrials.gov/show/NCT01730534) to evaluate the possibility that dapagliflozin may have a beneficial effect on the incidence of cardiovascular events in patients with T2DM and established cardiovascular disease or multiple risk factors.

Among some phase 2 and phase 3 trials in the dapagliflozin development program there was a small excess in event rates for male bladder cancer and female breast cancer over rates expected of the age-matched diabetic population [71, 78]. However, the imbalance was not statistically significant, and the diagnosis of all breast and bladder cancers within 1 and 2 years, respectively, of starting dapagliflozin and the wide biological heterogeneity of, in particular, the bladder cancers argue against a single causative agent. Updated data gathered from an additional 21 trials to November 2013 did not find any imbalance of malignancies in dapagliflozin-treated patients [78]. Nevertheless, although there are no indications of a statistically significantly increase in risk of cancer with dapagliflozin and it appears that early tumor diagnosis may be attributable to detection bias rather than as a result of a causal relationship, this issue, specifically regarding the risk of bladder cancer, will be further explored in ongoing dapagliflozin trials [71, 78]. Moreover, there have been no carcinogenicity or mutagenicity signals in the preclinical development program [65], and a recent study found that exposure of mice and rats to dapagliflozin for up to 2 years at levels greater than 100-fold and up to 186-fold human clinical exposure, respectively, did not increase bladder or mammary tumor rates, urinary bladder proliferative/preneoplastic lesions, or enhance tumor growth in murine models of human bladder transitional cell carcinoma, suggesting that dapagliflozin does not promote tumor growth [79].

In older patients (≥65 years), the incidence and nature of AEs was similar to that observed in the overall population and in patients younger than 65 years [71].

Although there was a higher incidence of fractures reported among dapagliflozin compared with placebo recipients in a study of T2DM patients with moderate renal impairment [35], there was no evidence for an imbalance in fracture rate of patients in the dapagliflozin and all control groups in the pooled safety data [71]. Furthermore, dapagliflozin did not affect markers of bone formation and resorption, or bone mineral density, at week 50 of a placebo-controlled study in 182 female and male patients with T2DM [80].

The generally favorable and predictable tolerability profile of dapagliflozin reported in this section is supported by the various meta-analyses and comprehensive reviews published to date [37–40, 71].

## Dapagliflozin in type 1 diabetes mellitus

Although dapagliflozin is currently only approved for the treatment of T2DM, studies of its efficacy and safety in T1DM are ongoing (Table 5). A pilot study describing 2 weeks of dapagliflozin treatment in 62 patients with T1DM showed dose-related reductions in glycemic variability, 24-h glucose and insulin requirement [81]. Patients with T1DM on stable insulin therapy were randomized to dapagliflozin 1, 2.5, 5, or 10 mg or placebo. Short-term tolerability was acceptable, and the pharmacokinetic profile was similar to that observed in studies in patients with T2DM, including dose-dependent increases in urinary glucose excretion. Changes suggestive of improved glycemic control were observed, which will be further explored in larger trials of longer duration.

**Table 5:** Trials of dapagliflozin in patients with type 1 diabetes mellitus currently registered on ClinicalTrials.gov

ClinicalTrials.gov identifier	Phase (status)	Study design	Dose	Main objectives
NCT02325206	I (Recruiting)	Randomized, placebo controlled, crossover	10 mg (single dose)	Safety and pharmacokinetics; degree of insulin dose reduction after 24-h
NCT01498185^a^	II (Completed)	Randomized, double-blind, placebo controlled	1, 2.5, 5, 10 mg × 14 days	Safety, change from baseline mean plasma glucose, pharmacokinetics and pharmacodynamics
NCT02268214	III (Recruiting)	Randomized, double blind, placebo controlled	5 or 10 mg × 52 weeks	Efficacy (change in HbA_1c_ at week 24; change from baseline insulin dose, metabolic parameters.
NCT02211742	IV (Recruiting)	Randomized, double-blind, crossover	10 mg × 3 days	Effect of dapagliflozin on fasting glucose homeostasis and postprandial glucose excursions in male patients

^a^Henry et al. [81]

## Conclusions

The SGLT2 inhibitors are a new class of antidiabetic agents with a unique, insulin-independent mechanism of action that depends only on plasma glucose and renal function. SGLT2 inhibitors offer benefits beyond glycemic control, including modest reductions in body weight and blood pressure and improved insulin sensitivity and β-cell function.

Dapagliflozin is an orally available SGLT2 inhibitor with an insulin independent mechanism of action and a lower risk of hypoglycemia than with conventional antidiabetic agents such as sulfonylureas and insulin. Dapagliflozin is effective as monotherapy or as an add-on to insulin or any other oral antidiabetic agent, and reduces both body weight and blood pressure. Clinical trials published to date have shown that dapagliflozin is effective and safe in patients with T2DM, both as monotherapy and in combination with other glucose-lowering agents, and recent data reporting 4-years’ clinical experience support these findings [56]. Moreover, recent findings have demonstrated a remarkably rapid action of dapagliflozin, with reductions in FPG levels within 1 week of treatment [77].

Dapagliflozin is effective in both early and late stages of T2DM, despite differences in disease status and concomitant medications, suggesting that the insulin-independent mechanism of action of dapagliflozin could make it a suitable treatment option throughout the different stages of clinical progression of T2DM.

Currently, dapagliflozin may be considered a second-line agent to treat patients with T2DM and has the potential to provide a first-line option, particularly for patients with contraindications or lack of glycemic control on metformin, the conventional first-line agent. Due to the frequency of genital infections, possibly related to induction of glycosuria, caution is indicated in patients prone to these types of infections. Long-term data on cardiovascular and mortality outcomes of dapagliflozin are awaited.

Dapagliflozin is also expected to be effective as an adjunct to insulin for the treatment of T1DM, and may be particularly appropriate in patients with T1DM treated with high doses of insulin or who need weight loss. However, clinical trials in patients with T1DM are still ongoing.

## Abbreviations

AE: adverse event
DPP-4: dipeptidyl peptidase-4
eGFR: estimated glomerular filtration rate
FPG: fasting plasma glucose
GLUT2: glucose transporter 2
HbA_1c_: glycosylated hemoglobin
HOMA: homeostasis model assessment
MACE: major adverse cardiac events
SGLT: sodium-glucose cotransporter
T1DM: type 1 diabetes mellitus
T2DM: type 2 diabetes mellitus
